# Plasma levels of leptin and soluble leptin receptor and polymorphisms of leptin gene -18G > A and leptin receptor genes K109R and Q223R, in survivors of childhood acute lymphoblastic leukemia

**DOI:** 10.1186/1756-9966-30-64

**Published:** 2011-06-01

**Authors:** Szymon Skoczen, Przemyslaw J Tomasik, Miroslaw Bik-Multanowski, Marcin Surmiak, Walentyna Balwierz, Jacek J Pietrzyk, Krystyna Sztefko, Jolanta Gozdzik, Danuta Galicka-Latała, Wojciech Strojny

**Affiliations:** 1 Department of Immunology, Chair of Clinical Immunology and Transplantation, Jagiellonian University Medical College ul. Wielicka 265, 30-663 Krakow, Poland; 2 Department of Pediatric Oncology and Hematology, Polish-American Institute of Pediatrics, Jagiellonian University Medical College ul. Wielicka 265, 30-663 Krakow, Poland; 3 Department of Clinical Biochemistry, Polish-American Institute of Pediatrics, Jagiellonian University Medical College ul. Wielicka 265, 30-663 Krakow, Poland; 4 Chair of Pediatrics, Polish-American Institute of Pediatrics, Jagiellonian University Medical College ul. Wielicka 265, 30-663 Krakow, Poland; 5 2nd Department of Medicine, Jagiellonian University Medical College, Krakow, Poland; 6 Department of Metabolic Diseases, Jagiellonian University Medical College, Krakow, Poland

## Abstract

**Background:**

Approximately 20% of children and adolescents in Europe are overweight. Survivors of pediatric acute lymphoblastic leukemia (ALL) are at increased risk of overweight and obesity. The purpose of this study was to assess leptin and leptin soluble receptor levels, as well as polymorphisms of selected genes in survivors of pediatric ALL, and the influence of chemo- and radiotherapy on development of overweight in the context of leptin regulation.

**Methods:**

Eighty two patients (55% males), of median age 13.2 years (m: 4.8 years; M: 26.2 years) were included in the study. The ALL therapy was conducted according to modified Berlin-Frankfurt-Munster (BFM; n = 69) regimen or New York (n = 13) regimen. In 38% of patients cranial radiotherapy (CRT) was used in median dose of 18.2Gy (m: 14Gy; M: 24Gy). Median age at diagnosis was 4.5 (m: 1 year; M: 16.9 years) and median time from completion of ALL treatment was 3.2 years (m: 0.5 year; M: 4.3 years). Patients with BMI ≥85 percentile were classified as overweight. Correlation of plasma levels of leptin and leptin soluble receptor, and polymorphisms of leptin gene -18G > A, leptin receptor genes K109R and Q223R, and the overweight status were analyzed in relation to gender, intensity of chemotherapy (high intensity vs. standard intensity regimens) and to the use of CRT.

**Results:**

Significant differences of leptin levels in patients treated with and without CRT, both in the entire study group (22.2+/- 3.13 ng/ml vs. 14.9+/-1.6 ng/ml; p < 0.03) and in female patients (29.9+/-4.86 ng/ml vs. 16.9+/-2.44 ng/ml; p = 0.014), were found. Significant increase of leptin levels was also found in overweight patients compared to the non-overweight patients in the entire study group (29.2+/-2.86 ng/ml vs. 12.6+/-1.51 ng/ml; p < 0.0001), female patients (35.4+/-6.48 ng/ml vs. 18.4+/-2.5 ng/ml; p = 0.005), and male patients (25.7+/-2.37 ng/ml vs. 6.9+/-0.95 ng/ml; p < 0.0001). Negative correlation was observed for plasma levels of soluble leptin receptor and overweight status, with significant differences in overweight and non-overweight patients, both in the entire study group (18.2+/-0.75 ng/ml vs. 20.98+/-0.67 ng/ml; p = 0.017) and in male patients (18.2+/-1.03 ng/ml vs. 21.8+/- 1.11 ng/ml; p = 0.038). Significant (p < 0.05) negative correlation was found between leptin and leptin receptor levels in the entire group (correlation coefficient: 0.393) and in both gender subgroups (correlation coefficient in female patients: -0.427; in male patients: -0.396).

**Conclusions:**

The prevalence of overweight in our cohort was higher than in general European population (31% vs 20%) and increased regardless of the use of CRT. Leptin and leptin receptor levels may be used as useful markers of high risk of becoming overweight in ALL survivors, particularly in females treated with CRT. Polymorphisms of leptin gene -18G > A and leptin receptor genes K109R and Q223R were not associated with overweight status in ALL survivors.

## Introduction

According to WHO, the prevalence of obesity in children in Europe has been rapidly increasing and it is expected to affect nearly 15 million children by 2010. Approximately 20% of adolescents and children are overweight. Moreover, 30% of those who are overweight actually fulfill the criteria of obesity. The epidemic of obesity results in substantial economic burden. It is currently responsible for 2-8% of healthcare costs and 10-13% of deaths in various parts of Europe [1]. Being overweight is a well-established risk factor of many chronic diseases, such as diabetes, hypertension and other cardiovascular diseases [2]. Survivors of pediatric acute lymphoblastic leukemia (ALL) are at substantially increased risk of developing obesity [3-5]. The most common explanations involve late effects of chemo-and radiotherapy, treatment with corticosteroids, altered life style, with prolonged periods of relative immobility and decreased energy expenditure. Leptin is a hormone synthesized mostly by white adipose tissue. Its structure is similar to cytokines. It plays a role of peripheral signal informing of the energy storage and thus participates in the long-term regulation of appetite and the amount of ingested food [6]. Plasma levels of leptin depend directly on adipose tissue mass and correlate with body mass index (BMI) [7]. Central and peripheral effects of leptin are mediated by leptin receptors located on cell surface [8]. Several isoforms of long form and short forms of leptin receptors are expressed in humans. The long form of leptin receptor is expressed primarily in the hypothalamus, and the short forms of leptin receptor are typical for peripheral tissues. Soluble leptin receptor is a unique form, which consists solely of extracellular domain of membrane leptin receptors [9]. By binding to this receptor, leptin delays its clearance from circulation [10]. This results in increased leptin levels and bioavailability and, as a consequence, potentiates its effect [11]. On the other hand, the plasma levels of soluble leptin receptors correlate with density of the leptin receptors on cell membranes [12]. In obese children with no comorbidities the levels of leptin are higher and the levels of soluble leptin receptor are lower than in non-obese children [13].

Therapy of ALL (chemo- and/or radiotherapy) may permanently modify the secretion of leptin and levels of leptin receptors [5]. Among the hereditary risk factors, the polymorphisms of leptin or leptin receptor genes provide a good opportunity to study the relationship between ALL and overweight status. To our knowledge there were no studies investigating polymorphisms of leptin and leptin receptor genes and their products in ALL survivors. Therefore, the aim of our study was to determine the polymorphisms of leptin and leptin receptor genes and plasma levels of leptin and leptin soluble receptors in survivors of childhood ALL. The study assessed the influence of chemo- and radiotherapy on leptin secretion and regulation and their effect on the development of overweight.

## Methods

The study group consisted of 82 subsequent patients aged 4.8 to 26.2 (median 13.2) years who have previously completed ALL therapy and were routinely seen at the outpatient clinic of the Department of Pediatric Oncology and Hematology, Polish-American Institute of Pediatrics, Jagiellonian University Medical College. The patients have started the ALL therapy from January 1985 through May 2005. The age at diagnosis of ALL was 1-16.9 (median 4.5) years. The ALL therapy was conducted according to subsequent revisions of modified BFM (69 patients) and New York (13 patients) regimens. In 31 patients cranial radiotherapy (CRT) was used according to the respective treatment regimens, in doses of 14 to 24 Gy (median 18.2 Gy). Second CRT (18 Gy) was applied in 1 patient. Details concerning ALL treatment protocols were published elsewhere [14-16]. Demographic and clinical data of the patients are provided in table 1. The median period between the end of ALL therapy and blood sampling in this study was 3.2 years (m:0.5 year; M:4.3 years).

**Table 1:** Patient characteristics

Feature	Total	CRT	No CRT
	
	Number of patients (%)		
Total	82 (100)	31(38)	51(62)

Gender:			
Female	37 (45)	16 (20)	21(26)
Male	45 (55)	15 (18)	30 (36)

ALL status:			
First complete remission	79 (96)	29 (35)	50 (61)
Relapses	3	2	1
CNS	1	1	0
Testes	2	1	1
BM + CNS	0	0	0

Intensity of protocol:			
High intensity	14 (17)	13 (16)	1 (1)
Standard intensity	68 (83)	18 (22)	50 (61)

Age at diagnosis(years)	1-16,9	1,9-13,7	1-16,9
Median	4,5	4,2	4,8

Age at study (years)	4,8-26,2	4,8-26,2	5,6-24,2
Median	13,2	17,7	11,4

Time from the start of	0,9-20,7	2,8-20,7	0,9-10,4
ALL treatment (years)			
Median	7,8	12,7	6,1

Time from completion of ALL treatment (years)	0,5-4,3	1,8-4,3	0,5-3,4
Median	3,2	2,7	3,2

ALL - acute lymphoblastic leukemia; CRT - cranial radiotherapy

Height and body weight measurements were performed by an anthropometrist. The Body Mass Index (BMI) and BMI percentile were calculated using online BMI calculators for patients ≤ 20 years [17] and patients > 20 years [18]. According to the terminology for BMI categories published in the literature [19], patients with BMI ≥85 percentile were classified as overweight.

### Biochemical tests

Fasting blood samples were collected for biochemical tests. The samples were collected in tubes containing EDTA and aprotinin and were immediately delivered to laboratory and centrifuged for 15 minutes at 3000 rpm. The plasma samples for peptide analysis were stored at - 80°C until the time of the assay. Levels of leptin and leptin soluble receptor were measured using commercially available EIA kits (R&D Systems, Inc., USA).

### Genotyping

All patients underwent genotyping, and in 77 cases good quality samples were available for further testing. Subsequently, DNA was extracted from peripheral leukocytes using QIAamp DNA Blood Mini Kit (QIAGEN, Germany). Appropriate DNA Blood Mini Kit (QIAGEN, Germany). Appropriate DNA fragments of leptin gene -18G > A, leptin receptor gene K109R and Q223R were amplified using PCR and analyzed using PCR-RFLP (Restriction Fragments Length Polymorphism), DHPLC (Denaturing High Performance Liquid Chromatography) or direct sequencing. The primer sequences are shown in table 2.

**Table 2:** Sequences of primers

Genetic polymorphism	Sequences of primers	Genotyping method used (restriction enzyme)
Leptin gene - 18G > A	tggagccccgtaggaatcgca tgggtctgacagtctcccaggga	PCR-RFLP (*Aci*I)

Leptin receptor gene - K109R	tttccactgttgctttcgga aaactaaagaatttactgttgaaacaaatggc	PCR-RFLP (*Hae*III)
Leptin receptor gene - Q223R	aaactcaacgacactctcctt tgaactgacattagaggtgac	PCR-RFLP (*Msp*I)

### Statistical analysis

The correlations of the genetic polymorphisms, biochemical test results, and overweight status were analyzed with regard to gender, intensity of chemotherapy (high intensity vs. standard intensity regimens) and to the use of CRT. Results were expressed as mean ± SEM. The data were analyzed by ANOVA followed by Scheffe's post hoc test. For between-group comparison of nonparametric variables Chi^2 ^test was used. Correlations between the variables were calculated using Pearson correlation. The P values < 0.05 were considered statistically significant. The statistical analyses were performed using the Statistica 8 software package (Stat Soft, Inc., USA).

Permanent Ethical Committee for Clinical Studies of the Medical College of the Jagiellonian University approved the study protocol. All parents, adolescent patients and adult patients signed written informed consent before blood sample collection. No patient refused participation in the study.

## Results

### Anthropometric evaluation

Median BMI percentiles at the time of ALL diagnosis and at the time of the study were 45.3 (m:0; M:99.6) and 65.5 (m:0.3; M:99.6), respectively. After the completion of ALL treatment BMI ≤ 10 percentile and ≥ 95 percentile was found in 9% and 13% of patients, respectively. At ALL diagnosis 21% of patients were classified as overweight (BMI ≥ 85), the respective proportion at the time of the present study was 31%. The prevalence of the overweight status at the time of ALL diagnosis/after ALL treatment in patients treated with and without CRT was 10%/23% and 20%/35%, respectively (table 3).

**Table 3:** Anthropometric evaluation

Patients	Total	CRT	No CRT
	
	Number of patients (%)		
Total	82 (100)	31 (38)	51 (62)

Gender:			
Female	37 (45)	16 (20)	21 (26)
Male	45 (55)	15 (18)	30 (36)

Overweight at ALL diagnosis	13 (16)	3 (10)	10 (20)

Overweight after ALL treatment	25 (31)	7 (23)	18 (35)

CRT - cranial radiotherapy

### Leptin and soluble leptin receptor

**Figure 1:**
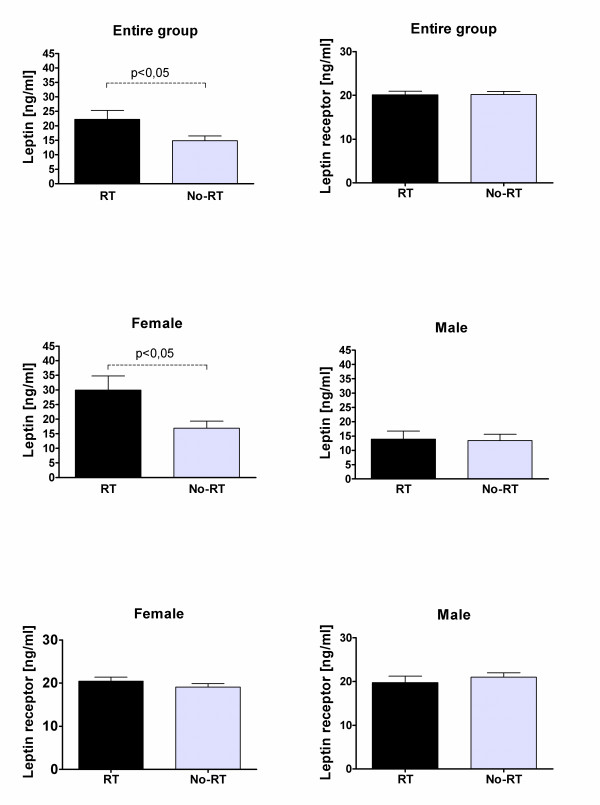
**Differences between leptin and leptin receptor levels in patients treated with and without CRT**.

**Figure 2:**
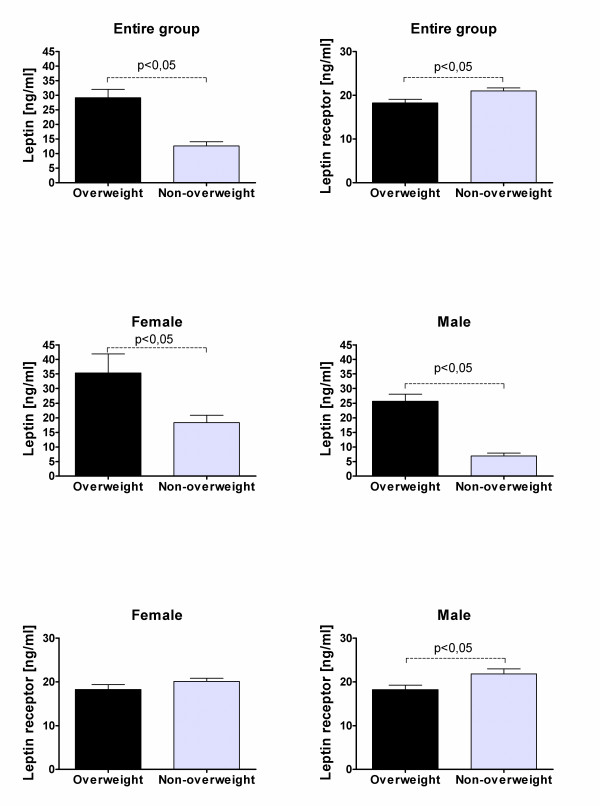
**Differences between leptin and leptin receptor levels in overweight and non-overweight patients**.

Significant differences were found between leptin levels in patients treated with and without CRT (figure 1) both in the entire study population (22.2+/- 3.13 ng/ml vs. 14.9+/-1.6 ng/ml; p < 0.03) and in female patients (29.9+/-4.86ng/ml vs. 16.9+/-2.44 ng/ml; p = 0.014). Significant increase of leptin levels was also found in overweight patients compared to the non-overweight subjects (figure 2) in the entire study group (29.2+/-2.86 ng/ml vs. 12.6+/-1.51 ng/ml; p < 0.0001), female patients (35.4+/-6.48 ng/ml vs. 18.4+/-2.5 ng/ml; p = 0.005), and male patients (25.7+/-2.37 ng/ml vs. 6.9+/-0.95 ng/ml; p < 0.0001).

**Figure 3:**
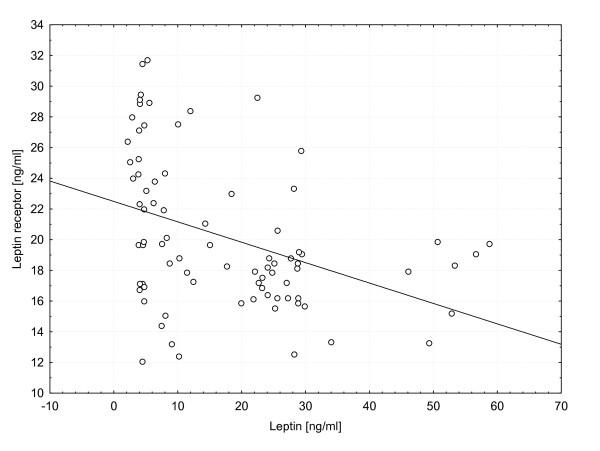
**Distribution of leptin receptor levels relative the leptin levels**.

Negative correlation was observed for soluble leptin receptor levels and body mass with significant differences in all overweight patients (18.2+/-0.75 ng/ml vs. 20.98+/-0.67 ng/ml; p = 0.017) as well as in overweight male patients (18.2+/-1.03 ng/ml vs. 21.8+/- 1.11 ng/ml; p = 0.038). Significant negative correlation (p < 0.05) was found between leptin and leptin receptor levels in the entire study group (correlation coefficient: 0.393) and in gender subgroups (correlation coefficient, female patients: -0.427; male patients: -0.396). In all subgroups two distinct clusters of leptin receptor levels (above and below 15 ng/ml) relative to leptin levels were observed (figure 3).

### Genotyping

The frequency of polymorphic homozygotes was assessed in the genotyped group. No significant correlation of the polymorphism of the leptin gene - 18G > A and the leptin receptor genes K109R and Q223R, and overweight status at ALL diagnosis and after ALL treatment was found. No statistically significant correlation between variants of the tested genes and intensity of ALL treatment, CRT and overweight status after ALL treatment was observed in the entire study group. The distribution of the tested polymorphisms in the study group is shown in table 4.

**Table 4:** Distribution of the of the tested polymorphisms in the study group

Genotyping group (n = 77)						
**Overweight**	**Leptin gene; -18G > A polymorphisms**		**Leptin receptor gene; K109R polymorphisms**		**Leptin receptor gene; Q223R polymorphisms**	
	
	**-18AA genotype**	**-18GG and -18GA genotypes**	**R/R genotype**	**K/K and K/R genotypes**	**R/R genotype**	**Q/Q and Q/R genotypes**

Yes	5	19	4	20	2	22

No	11	42	5	48	14	39

CRT (n = 30)						

Yes	0	7	2	5	1	6

No	3	20	1	22	5	18

No CRT (n = 47)						

Yes	5	12	2	15	1	16

No	8	22	4	26	9	21

CRT - cranial radiotherapy

## Discussion

Approximately 20% of adolescents and children in general European population are overweight, and 30% of these are obese [1]. In various studies the prevalence of obesity reported in survivors of ALL was 16 to 57%. An epidemic of pediatric and adult obesity in the developed countries is a well known phenomenon, but the studies also confirm that the prevalence of obesity in long-term survivors of ALL is substantially higher than in the general population [3]. In the cohort reported by Oeffinger *et al*. nearly half of the long-term survivors of childhood leukemia were overweight [20]. In our study the prevalence of overweight was 31%. Currently, 5 to 25% of children with ALL are classified to high risk groups and are treated with 18 Gy CRT. In the US approximately 25,000 to 30,000 long-term survivors of childhood ALL have a history of exposure to CRT. This represents 8 to 10% of all pediatric cancer survivors [21]. As radiotherapy is now spared to most patients with ALL and the doses applied in the high risk patients are lower (18 Gy), the clinical features of ALL survivors, that were common in the past, including short stature and obesity, are now less frequently seen. In our cohort CRT was used in 38% of patients, and the median dose was 18.2 Gy. Ross *et al*. suspected, that polymorphism of leptin receptor might influence obesity in female survivors of childhood ALL. Female survivors with BMI > 25 were more likely to be homozygous for the 223R allele (Arg/Arg) than those with BMI <25. Moreover, among females treated with CRT (≥20Gy), the patients who were homozygous for the 223R allele (Arg/Arg) had six times higher risk of BMI >25 than those with 223QQ or 223QR genotypes (Gln/Gln or Gln/Arg)[22].

In our study we have determined the polymorphisms of leptin and leptin receptor genes in pediatric population. Contrary to the results presented by Ross *et al*. we have not found any correlation of the selected polymorphisms of leptin and leptin receptor genes with overweight and the intensity of chemotherapy and/or CRT. We have not identified any oher studies revealing the influence of the polymorphisms of both leptin and leptin receptor genes on the metabolism of adipose tissue in survivors of childhood ALL. In our cohort we found highly significant increase in leptin levels in overweight patients in the entire study group and in gender subgroups. Negative correlation was found between leptin and soluble leptin receptor levels (in the entire study group and in male patients) suggesting negative feedback between those peptides. The same relationship was observed by other authors in children with uncomplicated obesity [12]. Significant increase of leptin levels in all patients treated with CRT and in female patients treated with CRT was observed. It was consistent with previous reports saying, that CRT causes accumulation of adipose tissue and that female patients are more affected than male patients [3,23,24]. As the soluble leptin receptor levels decrease, the clearance of leptin from circulation should be faster and its levels (and bioavailability) should be lower [10]. This is in discrepancy with higher incidence of overweight status in such patients. Because the plasma levels of soluble leptin receptors correlate with the density of leptin receptors on cell membranes [12], it is possible that after CRT involving the area of hypothalamus such density might decrease, thus reducing the inhibitory effect of the peripheral signal informing of the accumulation of body stores of energy. We cannot explain the presence of particular clusters of leptin receptor levels (above and below 15 ng/ml) relative to leptin levels (figure 3). Similar distribution of leptin levels and BMI was published by Arguelles *et al*.[25]. In the study by Janiszewski *et al*. the ALL survivors previously treated with CRT had higher absolute and relative (expressed per kg of fat mass) leptin levels than patients who were not treated with CRT. Females had higher absolute and relative leptin levels than males. Females treated with CRT had 60% higher fat mass than age-matched females from normal population [23,26]. The observation, that the history of CRT in ALL survivors is associated with increased plasma leptin levels suggests, that the pathogenesis of obesity may involve radiation-induced hypothalamic resistance to leptin. Alternatively, the elevated leptin levels may be a result of growth hormone (GH) deficiency, rather than manifestation of leptin resistance *per se *[27]. The history of CRT in ALL survivors is not only associated with accumulation of more abdominal fat, but causes its preferential accumulation in the visceral depot, possibly as a consequence of relative GH deficiency [23]. Transport of leptin from blood to CNS is mediated by leptin receptors localized on the endothelial cells of the blood-brain barrier. The dysfunction of these receptors might cause leptin resistance and obesity. The ventromedial hypothalamus is the site of leptin, ghrelin, neuropepeptide Y-2, and insulin receptors, which transduce peripheral hormonal afferent signals to control efferent sympathetic and vagal modulation, appetite, and energy balance [28]. High plasma leptin levels may be either a consequence of radiation-induced hypothalamic damage, or an effect produced by centrally induced GH deficiency, since hypothalamus is more sensitive to irradiation than pituitary [29]. As it was shown by Schwarz and Niswender, insulin and leptin receptors are located in key brain areas, such as the hypothalamic arcuate nucleus. In some cells of hypothalamus, leptin and insulin activate both JAK-STAT and PI3K signaling pathways. Additionally, both enzymes terminating leptin and insulin function -- SOCS3 and PTP-1B -- are expressed in the hypothalamus. Impaired receptor function (in the context of macrophage/inflammatory reactions) caused by radio/chemotherapy may be the reason of leptin resistance. The closed-loop leptin/insulin feedback makes the GH/insulin/leptin relations understandable [30,31]. According to Link *et al*. leptin might serve as a good marker for high risk of overweight/obesity, particularly in patients treated with CRT [5]. The lack of correlation of the tested genes and obesity in ALL survivors together with changes in leptin/soluble leptin receptor plasma levels suggest, that influence of the selected genetic polymorphisms was not very potent. It is possible that the treatment-related risk factors (i.e. CRT) have stronger impact. The small size of the study group makes more profound analysis difficult. The common additional explanation is the sedentary life style of ALL survivors. Almost 44% of adult survivors of childhood ALL are unlikely to meet the Centers for Disease Control and Prevention recommendations for physical activity and over 74% are less likely to be physically active [32]. When controlling for BMI, the ALL survivors treated with CRT were less likely to be physically active. Importantly, the ALL survivors with a confirmed history of previous GH therapy were 2.7 times more likely to be physically inactive than ALL survivors, who were at low risk for GH deficiency [33]. Again, it suggests hormone-dependent or regulatory peptide-dependent mechanism.

## Conclusions

1. The prevalence of overweight status in our cohort was higher than in general European population (31% vs 20%), and increased regardless of introducing of CRT.

2. Leptin and leptin receptor levels may serve as good markers for high risk of becoming overweight, particularly in female patients treated with CRT.

3. Polymorphisms of leptin gene -18G > A, and leptin receptor genes K109R and Q223R were not associated with overweight status in ALL survivors.

## List of abbreviations

ALL: acute lymphoblastic leukemia; BFM: Berlin - Frankfurt- Münster; BMI: Body Mass Index; CRT: cranial radiotherapy; DHPLC: Denaturing High Performance Liquid Chromatography; GH: growth hormone; RFLP: Restriction Fragments Length Polymorphism.

## Competing interests

The authors declare that they have no competing interests.

## Authors' contributions

SS designed and coordinated the study, collected the follow-up information, performed data analysis and drafted the manuscript, PT designed biochemical methods and performed biochemical analysis, performed data analysis and participated in drafting of the manuscript MB-M designed genotyping methods and performed genotyping, performed data analysis and participated in drafting of the manuscript, MS performed biochemical analysis, performed data analysis and participated in drafting of the manuscript, WB consulted the results and participated in drafting of the manuscript, JJP consulted the results and participated in drafting of the manuscript, KS consulted the results and participated in drafting of the manuscript, JG consulted the results and participated in drafting of the manuscript, DG-L consulted the results and participated in drafting of the manuscript, WS consulted the results, participated in drafting of the manuscript and critically revised the final version All authors read and approved the final version of the manuscript.
